# Inaccurate labelling practices in probiotic products: A regulatory shortfall in Accra, Ghana

**DOI:** 10.1371/journal.pone.0322194

**Published:** 2025-05-28

**Authors:** Mansa Fredua-Agyeman, Emmanuel Aduamah Larbi

**Affiliations:** Department of Pharmaceutics and Microbiology, School of Pharmacy, College of Health Sciences, University of Ghana, Legon, Accra, Ghana; University of Johannesburg, SOUTH AFRICA

## Abstract

The global market for probiotics has witnessed significant growth in recent years, driven by increased interest in their health benefits. However, amidst the substantial expansion of this industry, there are still challenges regarding their quality control, especially in the aspect of labelling accuracy, hence reliability. This study evaluated the labelling accuracy and availability of probiotic products in Ayawaso West Municipality, Accra, Ghana. Using a stratified sampling design, 120 pharmacies within the Municipality were randomly selected and assessed for availability of probiotics and labels scrutinized. Visual inspection of the product labels was carried out utilizing a probiotic product label assessment checklist developed as a guide. The checklist covered information including brand details, probiotic species/strains, cell quantity, ingredients, label claims, dosage instructions and expiration dates. The checklist was based on recommended labelling requirements by the Joint Food and Agriculture Organization of the United Nations and World Health Organization (FAO/WHO) and the Council for Responsible Nutrition (CRN) and International Probiotics Association (IPA). The findings from this study revealed 78.3% of pharmacies in the Ayawaso West Municipality offered probiotic supplements and/or probiotic foods. Disparities in recommended label requirements and the labels of probiotic products found within the Municipality was observed. Labelling issues were more prevalent in probiotic foods than supplements. Compliance for the probiotic supplements was 74% and 44% for the probiotic food. Common issues included lack of essential information like cell quantity or CFU (colony-forming-unit), lack of statement of specific probiotic strain contained in the product, lack of scientific references supporting health claims and un-existing bacteria names. The probiotic foods occasionally stated the genus, and some did not indicate species or strain contained in the product. The study sheds light on the gaps in labelling practices in the probiotic market in Ghana and advocates for greater compliance and proper regulation of probiotics.

## Introduction

Probiotics are defined as “live microorganisms that, when administered in adequate amounts, confer a health effect on the host” [1]. The probiotic market has experienced remarkable growth globally in recent years. The global market size of probiotics was valued at approximately USD 87.70 billion in 2023. It is estimated to grow at a compound annual growth rate (CAGR) of 14.1% from 2024 to 2030 [2]. The growth can be attributed to several factors. These include the rising consumer awareness of potential health benefits, evidence of health benefits of probiotic strains and the rising consumer interest in preventative healthcare. Additionally, there has been a higher demand for immune-boosting and natural products due to health crises like the COVID-19 [3–5]. The market’s expansion has led to a diverse range of probiotic products being available to consumers including different types of probiotic foods and supplements.

Unlike drugs where a regulatory compliant label which needs to be in accordance with given requirements by regulators of respective countries is mandatory for marketing, probiotic labels are not adequately regulated in several countries including Ghana [6–8]. Similarly, their efficacy and quality control are not particularly regulated. In countries where the regulatory framework for probiotics are more developed, for instance Canada, USA, Brazil, Japan, Australia, South Korea, South Africa among others, due to their diverse categorization, some probiotics might still not be subject to the same level of scrutiny as pharmaceutical drugs [8,9]. Pertaining to labelling of probiotics, guidelines issued by the Joint Food and Agriculture Organization of the United Nations and World Health Organization (FAO/WHO) for the evaluation of probiotics in food recommends that certain information are described on probiotic labels. The guideline recommends that the identity of probiotic present in a product are present on the label. It likewise recommends that the microbial species and where necessary, the strain identity based on the International Code of Nomenclature be indicated on the label since probiotic effect is strain specific. It also requires that the amount of viable cells present at the end of the shelf life be stated on the label as well as the minimum dosage and verifiable health claims be included [10,11]. The International Probiotics Association (IPA) in partnership with the Council for Responsible Nutrition (CRN) also developed a scientifically based voluntary guideline on probiotic labelling and emphasised the importance of providing specific information on probiotic product. The guideline state that labels should clearly indicate the identification of each microorganism contained in the product, identifying genus, species and strain. The claimed beneficial effects and the suggested serving size to obtain health benefits is recommended to be stated. The guideline also recommends that the label should state the appropriate storage conditions for the product. Also, the quantitative amount of live microorganisms expressed as colony forming units (CFUs) and the expiration date should be stated. For products containing multiple strain and or species, information on the total amount of microorganisms is suggested to be stated and reasonably, the amount of each species as well [12].

Although the provision of these information on labels may not necessarily be rigorously enforced by some national regulations, and may vary due to the absence of mandatory harmonized regulations, there is scientific agreement that such information is important to improve transparency and consistency in probiotic labelling [6,8]. They also enhance consumer understanding of probiotic products and enable well-informed choices regarding their usage while ensuring product safety and quality.

Despite the importance of accurate product labelling, challenges exist in the probiotic industry [6,7,13–17]. Inaccuracies in probiotic product labels including poor description of contents [13,14,16,18], misspelled bacterial names [16,18], unexisting bacteria names [18], unstated quantities of bacterial numbers [14,16,18], unsupported health claims [15] no expiration dates [16,18] have been reported.

In Accra, the capital city of Ghana, there is a growing interest in the use of probiotic products for health improvement and the prevention or treatment of various conditions. However, limited information is available regarding the accessibility, reliability and efficacy of probiotic products in Accra. This lack of information makes it challenging for consumers and healthcare professionals to make informed decisions about the selection and use of probiotic products. Moreover, there is no distinct regulatory framework for probiotics in Ghana, which raises concerns about the quality, consistency and safety of these products [19]. While there may be some regulatory oversight by the Food and Drugs Authority (FDA), the current approach to regulating probiotics in Ghana is generally less structured or specific compared to other countries with more established regulations.

The aim of this study is to analyse the accuracy of labels of probiotic product available for human use in Ayawaso West Municipality, one of the twenty-nine Metropolitan, Municipal and District Assemblies in Greater Accra Region, Ghana. This was to assess the compliance of probiotic product labels with international regulatory requirements and recommendations and to assess the availability of different types of probiotic products on the market.

## Methods

The study was conducted at the Ayawaso West Municipality in Accra. Ayawaso West Municipality is an urbanized area in the capital city of Ghana, similar to other areas in Greater Accra. The University of Ghana is located within this Municipality. A cross sectional survey by means of stratified random sampling of pharmacies across different suburbs of the Municipality was conducted between 11^th^ September 2023 and 18^th^ December 2023. Thus, the area was divided into suburbs (strata) and pharmacies were randomly selected. This approach aimed to capture variations in product availability across the different suburbs within the Municipality which included Legon, East Legon, East Legon Extension, South Legon, Airport Residential, Airport West Residential, Roman Ridge, Dzorwulu, North Dzorwulu, Agbelemkpe and Old Tesano. Although probiotics can be found in places other than pharmacies, pharmacies in Accra often offer a wider range of products, including beverages, yogurts, and confectionery, in addition to drugs and supplements. They are typically the first place consumers turn to when seeking probiotics.

Ethical approval was obtained from the University of Ghana, School of Pharmacy Ethics Committee (UGSOPEC) with approval number UGSOPEC/AC2022–2023/073. Written consent and approval of the pharmacist of participating pharmacies were obtained before product inspection.

Pharmacies across these different suburbs were targeted. A minimum required sample size of pharmacies was obtained using the formula:


$$n=\;\frac{N\;\times \;Z^{2}\;\times \;p\left(1\;-\;p\right)}{e^{2}}\;$$

Where:

n = sample size

Z = Z-score for a desired confidence level (i.e., 95% confidence level = 1.96)

p = estimated proportion of pharmacies with probiotic products (assumed to be 50%)

e = margin of error (set at 5% or 0.05)

The estimated population size (N) of pharmacies in Ayawaso West Municipality was obtained from the Pharmacy Council Ghana, Headquarters, Accra. The sample size was computed to be 120.

One hundred and twenty (120) pharmacies were surveyed. These were selected at random from each suburb. A probiotic label assessment checklist (Table 1) was developed based on recommendations from CRN and IPA and also from the FAO/WHO [10,12]. The checklist criteria included information such as brand/manufacturer, product name, probiotic strains, quantity of viable cells/CFU, list of included ingredients, label claims, dosage and usage instructions, expiration date, storage conditions and manufacturer information. Other information provided on the product labels were also captured.

**Table 1:** Probiotic product label assessment checklist.

Probiotic Product Label Assessment Checklist			
Criteria		Response	
		Yes	No
**Product Information**			
1.	Brand/Manufacturer		
2.	Product name		
**Probiotic Strain(s)**			
1.	Name(s) of probiotic species/strain(s)		
2.	Strain-specific quantity/CFU (if provided)		
**Ingredients**			
1.	List of ingredients		
2.	Any potential allergens or additives mentioned		
3.	Presence of any other active ingredients		
**Label Claims**			
1.	Specific benefits mentioned (e.g., immune support, gut health)		
2.	Any claims supported by scientific references		
**Dosage and Usage Instructions**			
1.	Recommended dosage per serving		
2.	Frequency of use		
**Expiry Date and Shelf Life**			
1.	Expiry date mentioned		
2.	Storage recommendations		
**Quality and Manufacturing Information**			
1.	Good Manufacturing Practices (GMP) certification mentioned		
**Additional Information**			
1.	Presence of prebiotics		
2.	Precautions or contraindications mentioned		
3.	Recommended age group		

Pharmacies, including pharmacies within shopping malls within the Municipality were visited to visually inspect the probiotic products. The products were grouped into supplement and food. Relevant information was collected from all probiotic products using the checklist and also captured in a spreadsheet. Descriptive statistics such as median, mean, frequencies and percentages were employed to analyze the collected data.

## Results and discussion

One hundred and twenty (120) pharmacies were surveyed in the Municipality. Out of the 120 surveyed pharmacies, 94 offered probiotic supplements and/or probiotic foods. East Legon, Airport Residential, Roman Ridge and North Dzorwulu had more than 80% of pharmacies with probiotic products. Between 61% and 80% of pharmacies in Legon, Agbelemkpe and Airport West Residential stocked probiotic products. Dzorwulu and East Legon Extension had 41% -60% of pharmacies with probiotic products whilst South Legon and Old Tesano had the least with 38% and 26% of pharmacies with probiotic products respectively. Thus, the study revealed a substantial prevalence of probiotic products across pharmacies in Ayawaso West Municipality, with around 78.3% of the surveyed pharmacies offering these products. This observation aligns with the global trend of increasing interest in probiotics, a trend that has been amplified by the COVID-19 pandemic [4]. However, the availability of the product across the various suburbs of the Municipality were different. The elements contributing to these differences are intricate. These could include the socio-economic status of the population of the suburbs which may relate with the stock variety or inventory levels of the pharmacies. Also, the level of education of the population and their acceptance of complementary and alternative medicine (CAM) as some people may view probiotic therapies as CAM [20]. Twenty-seven (27) different probiotic supplements and 18 probiotic foods were collected from the pharmacies in the Municipality for analysis.

**Fig 1:**
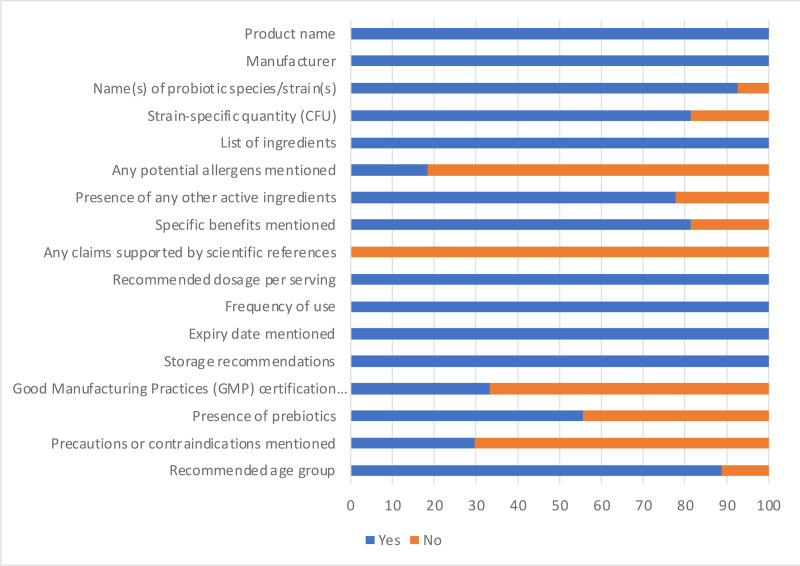
Compliance of the probiotic supplements to recommended label guidelines. Using the label assessment checklist (which included information such as name(s) of probiotic species/strain(s), strain-specific quantity/CFU, specific benefits, claims supported by scientific references, recommended dosage per serving etc.) responses were collected as yes (blue) or no (orange) and expressed as percentages on bar charts.

**Fig 2:**
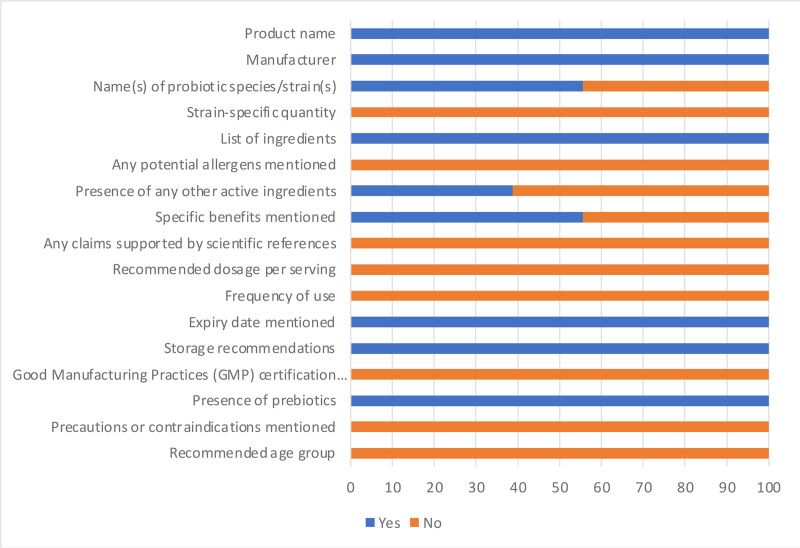
Compliance of the probiotic foods with recommended label guidelines. Using the label assessment checklist (which included information such as name(s) of probiotic species/strain(s), strain-specific quantity/CFU, specific benefits, claims supported by scientific references, recommended dosage per serving etc.) responses were collected as yes (blue) or no (orange) and expressed as percentages on bar charts.

Figs 1 and 2 summarize the responses of label criteria assessment of the probiotic supplements and foods respectively. Overall compliance for the probiotic supplements was 74% and 44% for the probiotic food. Certain aspects of label compliance, such as manufacturer information, storage recommendations and expiry date were generally met. However, critical aspects like strain-specific quantities/CFU counts and scientific references supporting health claims were often absent, which may raise questions about the substantiation of the purported benefits. Furthermore, the specific strains included in the products were mostly not declared even though the species included were indicated in more than 90% of the probiotic supplements. A distinct contrast emerged between probiotic supplements and foods. Notably, the majority of probiotic supplements demonstrated adherence to majority of the recommended label requirements. Misspelling/non-existing bacteria name was noted in a couple of the probiotic supplements. For instance, a product was labelled to contain *Lactobacillus* spores and another, *L. coagulans*. *Lactobacillus* species are non-spore forming. These are major labelling inaccuracies that need to be addressed by the manufacturers. Possibly, the manufacturers mislabelled *Bacillus* as *Lactobacillus*. Less than 20% of the supplements did not state the strain-specific quantity/CFU and specific benefits. The probiotic foods revealed a different situation. An insufficiency of essential information was observed. None of the probiotic food product displayed strain-specific quantity/CFU, scientific references supporting health claims, recommended dosage per serving, precautions/contraindications, or recommended age. A number of the probiotic food products did not state the specific probiotic strain or species contained in the product although the genus was provided for some. These labelling gaps, which have been documented in previous reports, highlight the persistent challenges in probiotic labelling and the need for mandatory harmonized regulations to promote safety, enhance consumer confidence, and contribute to the overall integrity of the probiotic market [9,16,21]. The inaccuracies in the labelling are especially important because of the unique nature of probiotic products. These products contain live microorganisms that can potentially cause diseases and negatively impact the gut microbiota particularly in immunocompromised individuals [22,23]. This is particularly significant given reports of antibiotic resistance of some strains [24,25]. For instance, some *Bacillus* spp. and *Enterococcus faecium* are known to possess toxicity and or antimicrobial resistance genes [26,27]. It is therefore critical that key information such as strains included, the amount/CFU needed to be consumed for health benefit and other label requirements are adhered to. Providing comprehensive and accurate information on labels not only guides consumers in making informed decisions but also assures product safety and alleviate uncertainties about product consumption.

The probiotic supplements were more compliant to recommended labelling requirement, with more products stating the species and quantity/CFU unlike the foods which commonly stated the genus without declaration of the cell quantity/CFU. The supplements were therefore further examined more thoroughly.

**Fig 3:**
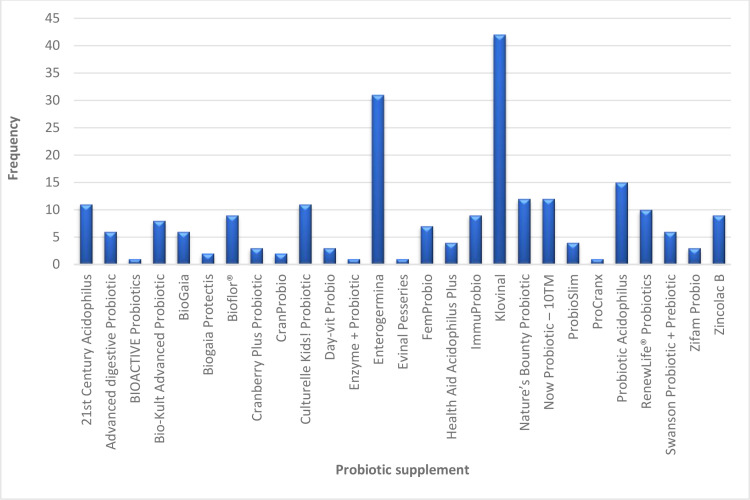
The frequency of different probiotic supplements in selected pharmacies in Ayawaso West Municipality.

Fig 3 represents a list of the probiotic supplements, each identified by its specific name, along with the frequency. Amongst the probiotic supplements, the most encountered product in the study was Klovinal, a vaginal pessary manufactured by Bliss GVS Pharma Ltd. (India) that contains *Lactobacillus* spores, according to the label. As mentioned previously, the genus *Lactobacillus* does not form spores. Potentially, the product contains *Lactobacillus* species or *Bacillus* spores or *Sporolactobacillus* which are entirely different genera, hence a serious labelling inaccuracy. The next commonly encountered product was Enterogermina^®^, a liquid product by Sanofi, which contains *Bacillus clausii* according to the label. Probiotic Acidophilus (Spring Valley), a caplet formulation containing *Lactobacillus acidophilus* was the third occurring product followed by Now Probiotic – 10^TM^ (Now Foods, USA) and Nature’s Bounty Probiotic, which is manufactured in USA. The least occurring probiotic supplements were Evinal pessaries (Ghana), ProCranx (USA), Enzyme + Probiotic (USA) and BIOACTIVE Probiotics (Bulgaria). More than 95% of the probiotic supplements were imported which emphasizes the country’s reliance on imported supply of medicine [28]. It also shows that probiotic products are widely available globally, with those in developed or Western markets also being accessible in developing markets, reflecting their broad distribution and consumer demand across different regions.

**Fig 4:**
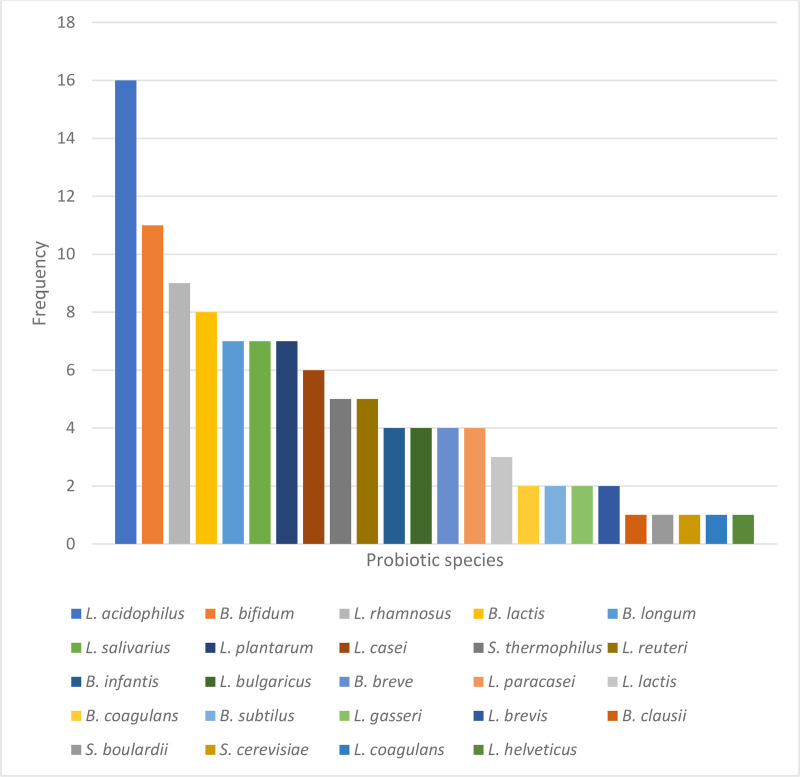
Species distribution in probiotic supplements evaluated.

The occurrence of probiotic species in the supplements analysed is shown in Fig 4. The commonest genera were *Lactobacillus* and *Bifidobacterium*. *Lactobacillus acidophilus* was the most occurring species with approximately 59.26% predominance*. Bifidobacterium bifidum* (40.74%) was the second most occurring species followed by *Lactobacillus rhamnosus* (currently reclassified as *Lacticaseibacillus rhamnosus*, 33.33%) and *Bifidobacterium lactis* (29.63%)*. Lactobacillus plantarum, Lactobacillus salivarus* and *Bifidobacterium longum* occurred at the same level (25.93%), then *Lactobacillus casei*, (recently reclassified as *Lacticaseibacillus casei,* 22.22%). *Streptococcus thermophilus* and *Lactobacillus reuteri* followed with similar occurrence (18.51%). The least occurring species included *Bacillus clausii*, Sacharomyces boulardii, Sacharomyces cerevisiae, L. coagulans^*^(wrongly labelled) and *Lactobacillus helveticus* (3.70%). The distribution of probiotic strains within the collected products which displayed a notable prevalence of certain strains, most prominently *L. acidophilus* is consistent with previous research [29–31]. *Lactobacillus acidophilus* is widely recognized to have probiotic effects such as regulation of microbiota balance, enhancement of gut health, enhancement of immune function, anticancer, antiaging and cholesterol lowering effects [29,32,33]. It has received considerable attention in research and development and is one of the most recommended probiotic bacteria for dietary use, having a huge commercial success [30,31]. *Bifidobacterium bifidum, B. lactis* and *L. rhamnosus* are also very common species. *L. rhamnosus* and *B. lactis* are among the top species which are very documented in probiotic research and it is no surprise they are contained in many of the products [34].

More than half of the probiotic supplements contained multi species. Multi-species products that contained more than 4 species contained *L. acidophilus*. Common combination/multi species included *L. acidophilus* with *B. bifidum* which occurred in 10 products, *L. acidophilus* with *B. lactis* which occurred in 8 products, *L. acidophilus* with *L. rhamnosus*, *L. acidophilus* with *L. salivarus*, *L. rhamnosus* with *L. plantarum* which occurred in 7 products respectively. This observation again highlights the prevalence of some species in the market space and also the appeal of multi species products which have the advantage of producing synergistic effects due to species/strain specificity of probiotic effects [35,36] although interactions have also been reported [35].

The probiotic supplements claimed to contain between 6 x 10^7^ CFU/dose to 5 x 10^10^ CFU/dose. Although the FAO/WHO guideline does not clearly state specific quantity, it has been recommended that products should contain 1 x10^9^ CFU per serving [1,37]. More than 60% of the probiotic supplements which stated their claimed quantity contained this recommended CFU or in excess. The products with highest claimed content of microorganisms included ImmuProbio and Femprobio both claiming to contain 5 x 10^10^ CFU although each contained 10 different and 12 different species respectively at different concentration. Products with the least amount of claimed content included the BioGaia products, Probiotic Acidophilus (1 x 10^8^ CFU) and Zincolac B (6 x 10^7^ CFU). More than half of the probiotic supplements were formulated as capsules (55.6%). Other dosage forms included tablets (14.8%), liquids (7.4%), pessaries (7.4%), gummies (3.7%) and powders. Capsules offer the advantage of avoiding harsh processing conditions, which means there is minimal loss of the live cells during manufacturing compared to other dosage forms. This benefit, along with the convenience and lower handling costs, likely makes capsules a preferred choice for packaging probiotics for manufacturers [38].

More than half of the assessed supplements had indication for gut health in adults. Only 7% were identified for use in children under 12 years of age. Thirty three (33) percent of the probiotic supplements were indicated for gastrointestinal health only, while 26% were indicated for gastrointestinal health and immune system support. About 3.7% were indicated for immune system support only, while 15% had claims in several categories. Majority of the products were predominantly recommended for oral use. It was observed that there was predominance of certain strains in products targeting specific functional benefit. For instance, products containing *L. acidophilus*, *L. rhamnosus, B. lactis* and *B. bifidum* were often associated with gastrointestinal (GI) health and GI health combined with immune support. *Lactobacillus acidophilus*, *B. lactis*, *B. bifidum* and *L. rhamnosus* are well-studied species known for their positive effects on gut health and immune modulation [39–41]. *Lactobacillus acidophilus* has been linked to improved digestion, reduced bloating and the prevention of pathogenic infections [42]. *Bifidobacterium lactis* and *B. bifidum* have demonstrated the ability to enhance the gut barrier function and regulate immune responses, particularly in cases of irritable bowel syndrome (IBS) and inflammatory bowel disease (IBD) [41]. *Lactobacillus rhamnosus* is recognized for its potential in preventing and treating diarrhea, enhancing mucosal barrier function and modulating the immune system [43].

## Conclusion

In conclusion, this study has revealed significant discrepancies between recommended label requirements and the actual labels found on probiotic products in Accra. Notably, labelling inaccuracies were more prevalent in probiotic foods compared to supplements. The shortcomings in labelling observed included non-existing bacterial names, lack of information on cell quantity or CFU, omission of specific probiotic strains contained in the product and absence of scientific references supporting health claims. Additionally, the data highlighted that probiotic products are widely distributed globally, with some of those available in developed Western markets also accessible in developing regions, indicating a broad distribution and high consumer demand.

The study illuminates the gaps in labeling practices in the probiotic market and advocates for greater compliance and proper regulation of probiotics in Ghana.

## Supporting information

S1 Data (XLSX)
